# Clinical Characteristics of 100 Patients With COVID-19-Associated Mucormycosis From a Tertiary Care Center in North India

**DOI:** 10.7759/cureus.25652

**Published:** 2022-06-04

**Authors:** Ravi Kant, Manjunath Totaganti, Bharati Mohan, Mukesh Bairwa, Prasan K Panda, Amit Tyagi, Amber Prasad, Yogesh Bahurupi

**Affiliations:** 1 Internal Medicine, All India Institute of Medical Sciences, Rishikesh, Rishikesh, IND; 2 Medicine, All India Institute of Medical Sciences, Rishikesh, Rishikesh, IND; 3 Otolaryngology, All India Institute of Medical Sciences, Rishikesh, Rishikesh, IND; 4 Microbiology, All India Institute of Medical Sciences, Rishikesh, Rishikesh, IND; 5 Community & Family Medicine, All India Institute of Medical Sciences, Rishikesh, Rishikesh, IND

**Keywords:** magnetic resonance imaging, computed tomography, diabetes mellitus, steroid use, covid-19-associated mucormycosis

## Abstract

Background

Fungal infection in patients with coronavirus disease 2019 (COVID-19) has emerged as a new challenge in healthcare facilities. This study aimed to describe the demographic and clinical characteristics of COVID-19-associated mucormycosis (CAM).

Methodology

This retrospective, single-center case series included patients who were hospitalized and diagnosed with COVID-19 and mucormycosis at the All India Institute of Medical Sciences, Rishikesh (North India) from April 15, 2021, onwards and last followed up on June 30, 2021. Demographic, clinical, laboratory, radiological, microbiological, pathological, and outcome data were then collected and analyzed.

Results

Of the 100 consecutive inpatients with CAM, 95 (95%) had diabetes mellitus. At the onset of illness, the most common manifestations were facial swelling (85%), eye swelling (83%), headache (68%), pain around the eyeball (67%), malaise (57%), and fever (50%). The most common organ involved on examination was the nose and paranasal sinus (96%), followed by the orbit (83%), palate (19%), and cranial nerves (7%). Pulmonary involvement was seldom observed (1%). Predominant pathological findings were the presence of aseptate hyphae (75%), necrosis (75%), angioinvasion (36%), and perineural invasion (2.6%). During the last follow-up, 13 patients died, with 11 (84.6%) having severe COVID-19 and two (15.3%) having moderate COVID-19.

Conclusions

Steroid use and diabetes mellitus are the significant risk factors of CAM. Patients with CAM usually present with face/eye swelling with radiological involvement of the nose and sinus and may die because of severe COVID-19.

## Introduction

In 2019, a new viral disease emerged from Wuhan, China, caused by severe acute respiratory syndrome coronavirus 2 (SARS-CoV-2). This disease is known as coronavirus disease 2019 (COVID-19), which has resulted in a pandemic according to the World Health Organization, affecting more than 185 million people worldwide, with over 4.01 million deaths as of July 9, 2021. Meanwhile, in India, the Ministry of Health and Family Welfare reported 30 million COVID-19 cases, with 2.9 million who recovered and more than 4 lakh who died [1].

According to the International Diabetes Federation, India has the second largest number (77 million) of adults with diabetes mellitus (DM) worldwide [2] In India, DM is the most common risk factor of mucormycosis, accounting for 50% of all mucormycosis cases. In a recent nationwide multicenter study in India, 57% of patients with mucormycosis had uncontrolled DM and 18% had diabetic ketoacidosis [3].

A sudden outburst of invasive fungal infection was reported in patients of COVID-19 from Egypt [4]. Moreover, the incidence of acute invasive fungal rhinosinusitis is higher in patients post-COVID-19 than in those without, especially in patients with an immunocompromised state, DM, renal disease, and liver dysfunction [5].

COVID-19 causes immune dysregulation in the body by reducing the numbers of CD4+T and CD8+T cells and some other alterations in innate immunity, leading to secondary fungal infections in the form of invasive rhinosinusitis. Thus, immune dysregulation caused by COVID-19 is associated with a significant incidence of secondary infections, both bacterial and fungal. The use of steroids and broad-spectrum antibiotics for COVID-19 treatment may also result in the development/exacerbation of pre-existing fungal diseases [6,7].

Many individual cases of mucormycosis related to COVID-19 and DM have been published [8,9]. Recently, Moorthy et al. reported 18 cases from India, suggesting a significant increase in the incidence of angioinvasive maxillofacial fungal infections in patients with DM (A4) treated for SARS-CoV-2 with a strong association with corticosteroid use. Out of 18 patients, 12 suffered from vision loss and seven underwent orbital exenteration. Mucormycosis was found in 16 patients, aspergillosis in one patient, and mixed fungal infection in one patient. Furthermore, 11 survived, six died, and one was lost to follow-up. The incidence of DM was significantly higher (p = 0.03) among patients with COVID-19-associated mucormycosis (CAM). A significantly higher number (p = 0.0013) of patients received steroids at some point during the treatment [10].

Currently, amphotericin B and other limited antifungals, along with surgery, are the preferred strategy for managing mucormycosis. This case series aimed to describe the demographic, clinical, laboratory, radiological, microbiological, pathological, and outcome characteristics of 100 patients with CAM.

## Materials and methods

Ethical consideration

This case series was part of a project entitled Disease Profile of COVID-l9 (DPC-I9), which included patient follow-up at a tertiary institute in India, and was approved by the Institutional Ethical Committee (approval number: AIIMS/IEC/20/817).

Data collection

This study included 100 patients with CAM admitted between May and June 2021 to the All India Institute of Medical Sciences, Rishikesh located in North India. Consent was taken from all participants. Data on baseline demographics, presenting signs and symptoms, disease characteristics, microbiological and radiological findings, treatments, and mortality outcomes were collected for the analysis. The extent and severity of the disease were determined through detailed history taking, comprehensive otorhinolaryngological examination, ophthalmologic evaluation, and neurological examinations. All healthcare staff concerned in patient care followed the COVID-19 protocol and used full personal protective equipment including N95 masks, gowns, gloves, face shields, and safety goggles. Routine blood tests, including complete blood counts, blood sugar, liver function tests, kidney function tests, glycated hemoglobin, and serum ferritin levels were performed. In brief, information such as demographic data, medical history, exposure history, underlying comorbidities, symptoms, signs, laboratory findings, imaging findings, and treatment measures during the hospital stay was collected. Acute kidney injury was identified according to the definition by Kidney Disease: Improving Global Outcomes [11].

Imaging and microscopy

**Figure 1:**
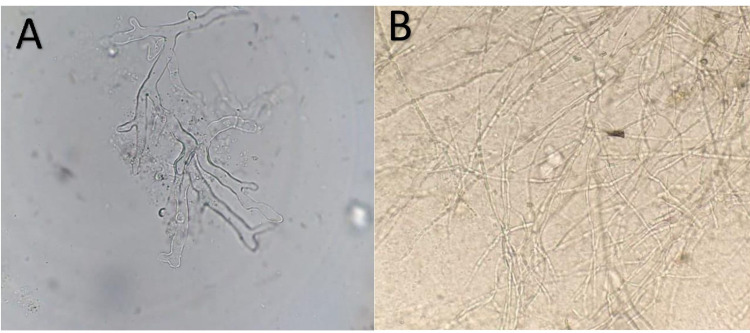
Potassium hydroxide mount of a biopsy sample showing (A) broad, pauciseptate, ribbon-like, perpendicular, and branching hyaline hyphae and (B) thin, acute-angle, and branching hyaline hyphae.

**Figure 2:**
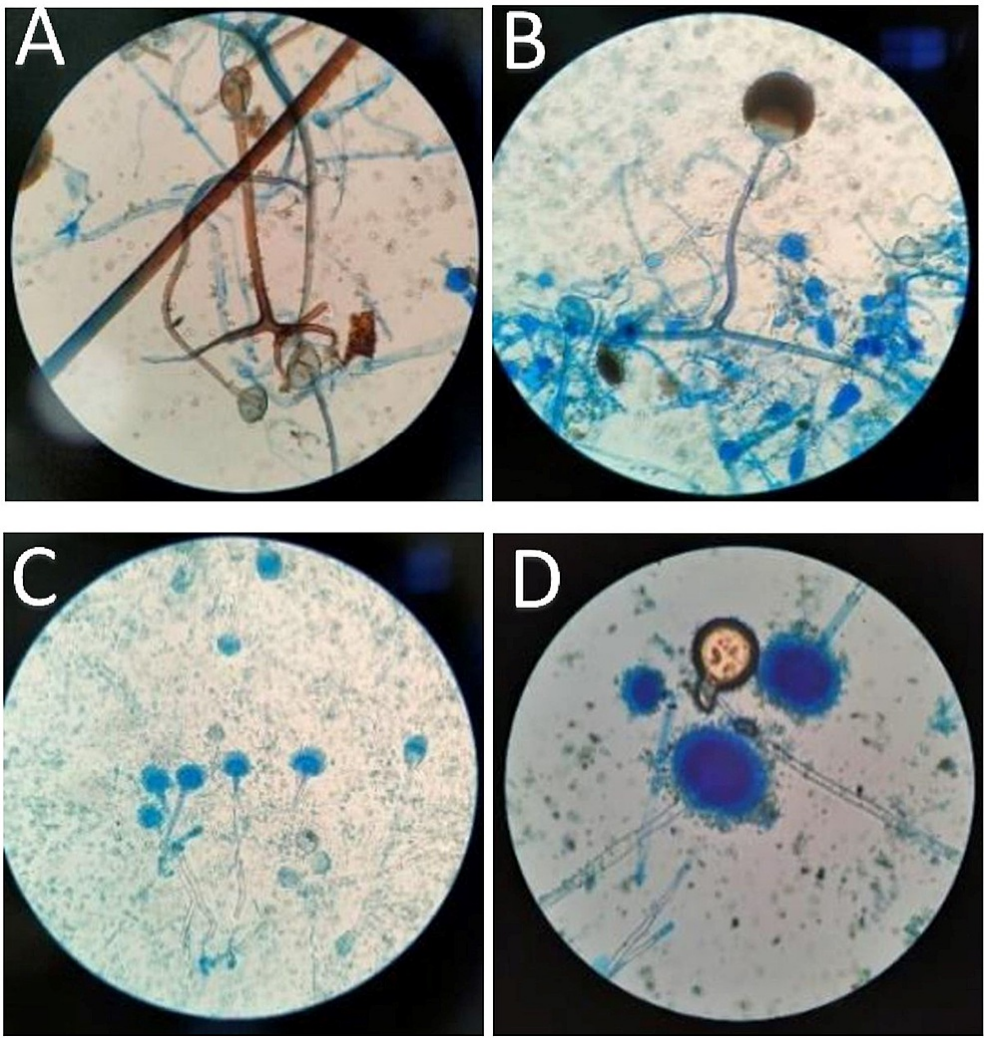
Lactophenol cotton blue mount showing (A) Rhizopus arrhizus, (B) Rhizomucor spp., (C) Aspergillus flavus, and (D) Aspergillus fumigatus.

Microscopy (direct and on histopathology) and culture, which are the cornerstones of diagnosis, were obtained. As per the treatment plan, the workup also included the scraping of exudates from the nasal cavity and/or paranasal sinuses, hard palatal lesions, and sinus material, along with a biopsy of the extracted tooth socket area, and the endoscopic collection of debrided tissue for biopsy (Figures 1, 2).

The extent of the disease was identified through radiological investigations, including computed tomography (CT) and/or magnetic resonance imaging (MRI) of paranasal sinuses, orbit, brain, and thorax. Retinal involvement was assessed by fundus imaging and optical coherence tomography.

The patients underwent medical and/or surgical management depending on the extent of the disease. A multidisciplinary team composed of an otorhinolaryngologist, an ophthalmologist, a neurosurgeon, and an internist coordinated the management.

Statistical analysis

Data were transported to Microsoft Excel and were analyzed using the SPSS version 23 (IBM Corp., Armonk, NY, USA). Categorical variables are expressed as frequency and percentages, whereas continuous variables are presented as mean, median, mode, and standard deviation.

## Results

Presenting characteristics

The study group comprised 100 inpatients with confirmed CAM (Table 1), with a mean age of 50.53 years, slight male predominance, and rural residence predominance. DM was the most common comorbidity, and approximately one-third of the patients were diagnosed in the hospital recently. Most of them had poor glycemic control, as indicated by their glycated hemoglobin. Of these 100 patients, approximately 85% had moderate-to-severe COVID-19 according to the WHO clinical classification. Furthermore, 84 patients had a history of oxygen therapy, 81 had a history of steroid intake, patients received remdesivir, and none received monoclonal antibodies (dose and duration of the therapy were not known). In addition, seven patients received a single dose of vaccine, three completed the vaccination schedule, and 90 were unvaccinated against COVID-19.

**Table 1:** Site of involvement on imaging and outcomes.

Paranasal sinus involvement on imaging	n = 100
Orbital involvement in imaging	99
Cavernous sinus involvement	85
Cerebral	11
Pulmonary	4
Outcomes	
Death	13
Discharged	9
In hospital	78

**Figure 3:**
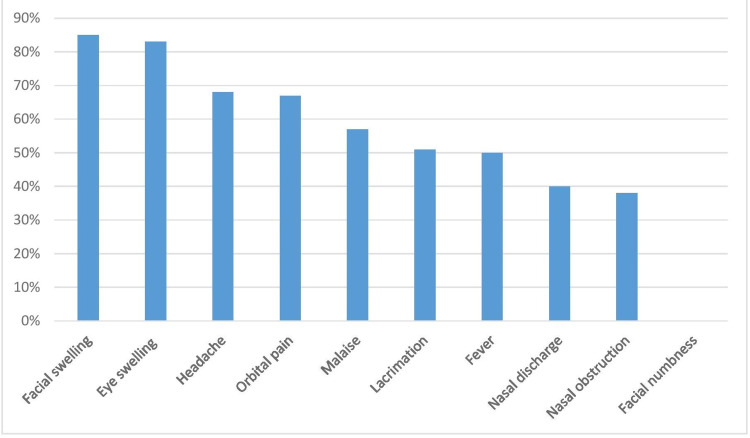
Bar diagram showing the symptoms of 100 patients with COVID-19-associated mucormycosis. COVID-19: coronavirus disease 2019

At the onset of illness, the most common and initial manifestations were facial and periorbital swelling and constitutional symptoms. Toothache and epistaxis were rarely observed (Figure 3).

On examination, the most common organ involved was the nose and paranasal sinuses, followed by the orbit; pulmonary involvement was infrequent in our case series (Figure 3, Table 1).

Vital signs and laboratory parameters

The baseline and demographic characteristics of patients included in this study are presented in Table 2.

**Table 2:** Baseline and demographic characteristics of patients with CAM (n = 100). CAM: coronavirus disease 2019-associated mucormycosis; COPD: chronic obstructive pulmonary disease; DBP: diastolic blood pressure; DM: diabetes mellitus; SBP: systolic blood pressure; SD: standard deviation

Age, in years (mean ± SD)		50.53 ± 11.14
Sex	Male	59
	Female	41
Residence	Urban	48
	Rural	52
Infection type	Nosocomial	49
	Community-acquired	51
Comorbidities	DM	95
	Systemic arterial hypertension	15
	Chronic kidney disease	3
	Cerebrovascular accident	1
	COPD	1
COVID-19 infection category	Mild	15
	Moderate	37
	Severe	48
Steroid use		81
Oxygen therapy		84
Diabetic ketoacidosis		12
Heart rate, per minute (mean ± SD)		84 ± 14
SBP, in mmHg (mean ± SD)		126 ± 11.05
DBP, in mmHg (mean ± SD)		79.6 ± 7.1

Most of the patients had leukocytosis with no organ involvement. Baseline investigations are shown in Table 3.

**Table 3:** Laboratory findings of patients with CAM. CAM: coronavirus disease 2019-associated mucormycosis; HbA1c: glycated hemoglobin; SD: standard deviation

	Reference range	Mean ± SD
White blood count, ×10^9^	4.00–10.00	12.87 ± 50.86
Neutrophils, %	40.00–80.00	77.9 ± 13.8
Lymphocytes, %	20.00–40.00	13.3 ± 10.3
Platelet count, ×10^9^	150.00–450.00	258.46 ± 117.67
Alanine aminotransferase, U/L	≤45	46.0 ± 56.15
Aspartate aminotransferase, U/L	0–40	32.8 ± 38.51
Total bilirubin, mg/dL	0.30–1.20	0.51 ± 0.23
Creatinine, mg/dL	0.5–1.0	1.09 ± 0.82
Blood urea, mg/dL	13–43	42.4 ± 30.30
Ferritin, ng/mL	10.00–291.00	534.9 ± 724.20
Random blood sugar, mg/dL	70–200	225 ± 92.56
HbA1c, %	<5.7	10.47 ± 2.8

The most common organism isolated from the cultures was Rhizopus arrhizus, followed by no growth in 31.5%, combined Rhizopus arrizhus in 7.8%, and Aspergillus flavus (Table 4). The histopathology report of 76 patients revealed that the majority had aseptate hyphae (57, 75%) and necrosis (57, 75%), followed by angioinvasion in 28 (36.8%) and perineural invasion in two (2.6%).

**Table 4:** Microbiological and histopathology characteristics of patients with CAM. CAM: coronavirus disease 2019-associated mucormycosis

Fungal culture	(n = 76)	Histopathology finding	(n = 76)
Rhizopus arrhizus	39.4%	Aseptate hyphae	75%
No growth	31.5%	Necrosis	75%
R. arrizhus and Aspergillus flavus	7.8%	Angioinvasion	36%
A. flavus	6.5%	Perineural invasion	2.6%
R. arrihizus and A. fumigatus	2.2%		
A. fumigatus	2.2%		
Others	10.4%		

Interventions

Medical management with antifungals was the mainstay of treatment. Approximately 95 patients received different formulations of amphotericin B; one received posaconazole based on the availability and affordability of the patients.

Surgical intervention was the solution to hasten recovery from the disease. Most of the patients underwent endoscopic debridement and maxillectomy (Table 5). The adverse effects of the medical management were also monitored and recorded.

**Table 5:** Treatment of patients infected with mucormycosis and treatment-related complications.

Drugs	Number of patients
Amphotericin-B lyophilized	95
Amphotericin-B lipid complex	1
Amphotericin-B liposomal	3
Posaconazole	1
Functional endoscopic sinus surgery	60
Maxillectomy	49
Orbital exenteration	13
Conservative management	11
Septoplasty	3
Complications related to treatment	
Drug-induced fever	28
Drug-induced chills	49
Hypokalemia	95
Acute kidney injury	35

Outcomes

Out of 100 patients, nine patients got discharged and 13 succumbed to death, with 10 (76.9%) males and three (23.07%) females. In total, 11 (84.6%) patients had severe COVID-19 and two (15.3%) had moderate COVID-19. All patients who expired had history of receiving steroids 13 (100%) for COVID-19 illness, and the remaining patients were still in the hospital receiving the treatment.

## Discussion

The second wave of COVID-19 in India led to considerable morbidity and mortality, with CAM as the new entity. In our case series, the predominant demographic risk factors of CAM were male sex, older age, and rural residence. DM (associated with diabetic ketoacidosis) demonstrating poor glycemic control was the most common and significant risk factor of CAM. The severity of COVID-19 infection and the usage of steroid and oxygen therapy were significant risk factors in our cases. In addition, leukocytosis was a significant laboratory abnormality. Rhino-orbital mucormycosis was also predominant. In brief, CAM is a fatal disease that needs long-term therapy. In this case series, 13 patients died because of CAM, and the remaining 77 were still in the hospital for long-term therapy.

Mucormycosis (sometimes called zygomycosis) is a rare but serious fungal infection caused by a group of molds called mucoromycetes. These fungi live throughout the environment, particularly in soil and decaying organic matter, such as leaves, compost piles, or rotten wood [12]. In this case series, this disease was more common in patients living in rural areas than in those in urban areas, possibly attributed to a better and clean environment in urban areas. In a systematic review of CAM, males were at a higher risk than females [13], consistent with our findings. Other risk factors of mucormycosis include DM (especially with diabetic ketoacidosis), cancer, organ transplant, stem cell transplant, neutropenia, long-term corticosteroid use, injection drug use, iron overload or hemochromatosis, surgery-induced skin injury, burns, wounds, and prematurity and low birthweight (for neonatal gastrointestinal mucormycosis) [14,15]. In our case series, DM and poor glycemic control were the significant risk factors of CAM, consistent with the abovementioned studies and other studies [16]. COVID-19 has been associated with a myriad of infections and complications, and one of them is CAM, which emerges as the deadliest complication. Patients with COVID-19 symptoms may offer a conducive environment for Mucorales growth. Patients with DM are more prone to develop COVID-19 and mucormycosis. Possible mechanisms of CAM include reduced viral clearance, decrease in T cell function, cytokine storm, and associated immunosuppression [17].

Patients with COVID-19 were treated with corticosteroids, which reduce white blood cells and T cells, thereby further decreasing immunity. Steroids also lead to hyperglycemia, which acts as a risk factor of mucor growth. COVID-19 also affects iron metabolism, resulting in increased levels of ferritin and reactive oxygen species. Cytokine storm also increases free iron in the circulatory system, leading to mucor growth [18].

This case series found facial and periorbital swelling, orbital pain, headache, and malaise as the most common presenting symptoms, similar to the study of Kursun et al. [19]. In another study, prominent complaints were fever, facial edema, orbital swelling, facial pain, and nasal obstruction [20].

Moreover, imaging detected the involvement of paranasal sinus, orbital, cranial nerve, palate, and cavernous sinus, similar to the study of El-Khily et al. [3]. CT can reveal the involvement of organs, typically showing mucosal thickening, edema, and infarcts. CT has an advantage over MRI because it can identify bone involvement and necrosis. However, MRI of the brain is often used in cerebral mucormycosis because it can detect early neural involvement. Furthermore, the head and surgeries show a very high risk for COVID-19 infection [21]. Surgical debridement is the key treatment approach for invasive fungal infection. In this case series, functional endoscopic sinus surgery, maxillectomy, and orbital exenterating were performed in 56, 30, and five patients, respectively, similar to a previous study. This previous study was written as a thesis, never in a manuscript, describing similarities or dissimilarities [22]. The surgical interventions of CAM with their respective side effects should also be investigated in detail.

In previous studies, liposomal amphotericin B was the only effective therapy for invasive mucormycosis, and posaconazole is an alternative drug for salvageable therapy. Liposomal amphotericin B is currently used as a first-line treatment at a dose of 5-10 ng/kg per day. Lipid complex formulation is a good alternative in patients without central nervous system (CNS) involvement. Serum creatinine elevation is reversible. However, the duration of therapy is uncertain; it is usually continued until immunosuppression is reversed or the disease is cleared on imaging [23,24].

Despite the aggressive surgical and medical management protocol, the mortality and morbidity of invasive fungal infection remain high, ranging from 18% to 80% [25], and they are very high in patients with CNS involvement. Although aggressive management was provided, 13 of our patients died, and others were still under treatment.

Regarding research limitations, this study was only conducted in one center, a tertiary care hospital in North India, with a relatively small number of cases with short-term follow-ups.

## Conclusions

In this study, DM and steroid use were the main risk factors of CAM. Early diagnosis and aggressive management with surgical debridement and antifungal treatment lead to a better prognosis, reducing the overall mortality and morbidity.
